# SCpubr: a user-friendly R-package for generating publication-ready visualizations of single-cell transcriptome analyses

**DOI:** 10.1093/bioadv/vbag151

**Published:** 2026-06-20

**Authors:** Enrique Blanco-Carmona, Marcel Kool

**Affiliations:** Division of Pediatric Neurooncology, Hopp Children’s Cancer Center (KiTZ), Heidelberg, Germany; German Cancer Research Center (DKFZ), German Cancer Consortium (DKTK), Heidelberg, Germany; Division of Pediatric Neurooncology, Hopp Children’s Cancer Center (KiTZ), Heidelberg, Germany; German Cancer Research Center (DKFZ), German Cancer Consortium (DKTK), Heidelberg, Germany; Princess Máxima Center for Pediatric Oncology, Utrecht, the Netherlands; University Medical Center Utrecht (UMCU), Utrecht, the Netherlands

## Abstract

**Motivation:**

Single-cell RNA sequencing (scRNA-seq) is now a core technology for resolving cellular heterogeneity in complex samples, and standard analysis workflows produce a wide range of outputs, each requiring tailored visualization. To support this, a wide range of analysis tools have been developed, many of which offer built-in visualizations but leave further customization to the user. Researchers who run standard single-cell workflows in R, often experimental biologists with a working knowledge of Seurat and ggplot2, still spend considerable effort converting analytical outputs into figures that meet journal standards.

**Results:**

We present SCpubr, an R package that provides concise function calls for generating high-quality, publication-ready visualizations commonly used in single-cell transcriptome analyses.

**Availability and implementation:**

SCpubr is available on CRAN (https://cran.r-project.org/package=SCpubr), with source code accessible on GitHub (https://github.com/enblacar/SCpubr).

**Supplementary information:**

Supplementary figures are available at Bioinformatics Advances online. Extensive documentation and tutorials are available via the GitHub Pages site (https://enblacar.github.io/SCpubr-book/). The complete analysis code used to generate all figures in this publication, along with the full R session information and instructions for obtaining the raw input data, is available in GitHub (https://github.com/enblacar/SCpubr-manuscript).

## 1 Introduction

Over the past decade, single-cell transcriptomics has advanced rapidly, with substantial efforts dedicated to establishing gold-standard guidelines for data analysis (Luecken and Theis 2019, Slyper *et al*. 2020, Heumos *et al*. 2023). This progress has driven the development of numerous software tools aimed at streamlining the analysis process. Notable examples include analysis frameworks such as Seurat (Hao *et al.* 2023), scanpy (Wolf *et al*. 2018), as well as data storage frameworks such as SingleCellExperiment (Amezquita *et al*. 2020) within the Bioconductor ecosystem, which have in turn enabled the creation of more than a thousand framework-dependent tools tailored to specific single-cell transcriptome analyses (Zappia and Theis 2021).

In parallel, specialized software for data visualization has emerged, reflecting the central role of effective visualization in scientific communication. Beyond the aforementioned frameworks, tools such as scCustomize (Marsh *et al*. 2024), LotOfCells (Gonzalez-Velasco 2024), plot1cell (Wu *et al*. 2022), dittoSeq (Bunis *et al*. 2021) or the iSEE shiny app (Rue-Albrecht *et al*. 2018), have been developed in order to generate data visualizations (Table 1, Table S1, available at supplementary material  *Bioinformatics Advances* online). Expanding on this, we present SCpubr, an R package providing a comprehensive plotting framework spanning six data visualization categories, with colorblind-safe support and an aesthetical focus.

**Table 1:** Systematic comparison of the scope, intended use, input structure, design philosophy, and availability of different visualization types between SCpubr (v3.0.0), Seurat (v5.4), dittoSeq, scCustomize, and LotOfCells.

Feature	SCpubr	Seurat	dittoSeq	scCustomize	LotOfCells
*Scope*	Publication-ready plot wrappers	End-to-end scRNA-seq analysis	Colorblind-friendly visualization	Helper utilities and enhanced plots	Cell-composition statistics and plots
*Intended use*	Figure-ready visualizations	Primary workflow toolkit	Accessible plots across modalities	Quality of Life extension of Seurat	Cell composition analysis
*Input structure*	Seurat Object	Seurat Object	SingleCellExperiment and Seurat Object	Seurat Object	SingleCellExperiment or Seurat Object
*Design philosophy*	Aesthetical data visualizations	Comprehensive, self-contained	Accessibility-first	Pragmatic Seurat extension	Single research question focus
*Visualization types*	See Table S1, available at supplementary material *Bioinformatics Advances* online				

SCpubr is designed for users who already run standard Seurat-based workflows and have basic familiarity with R. SCpubr provides a suite of high-quality, publication-ready visualizations through concise function calls, covering common results from single-cell transcriptomic analyses. SCpubr does not aim to replace specialized analytical tools, but instead focuses on streamlining the generation of publication-quality figures for standard single-cell outputs.

## 2 Tool description

**Figure 1:**
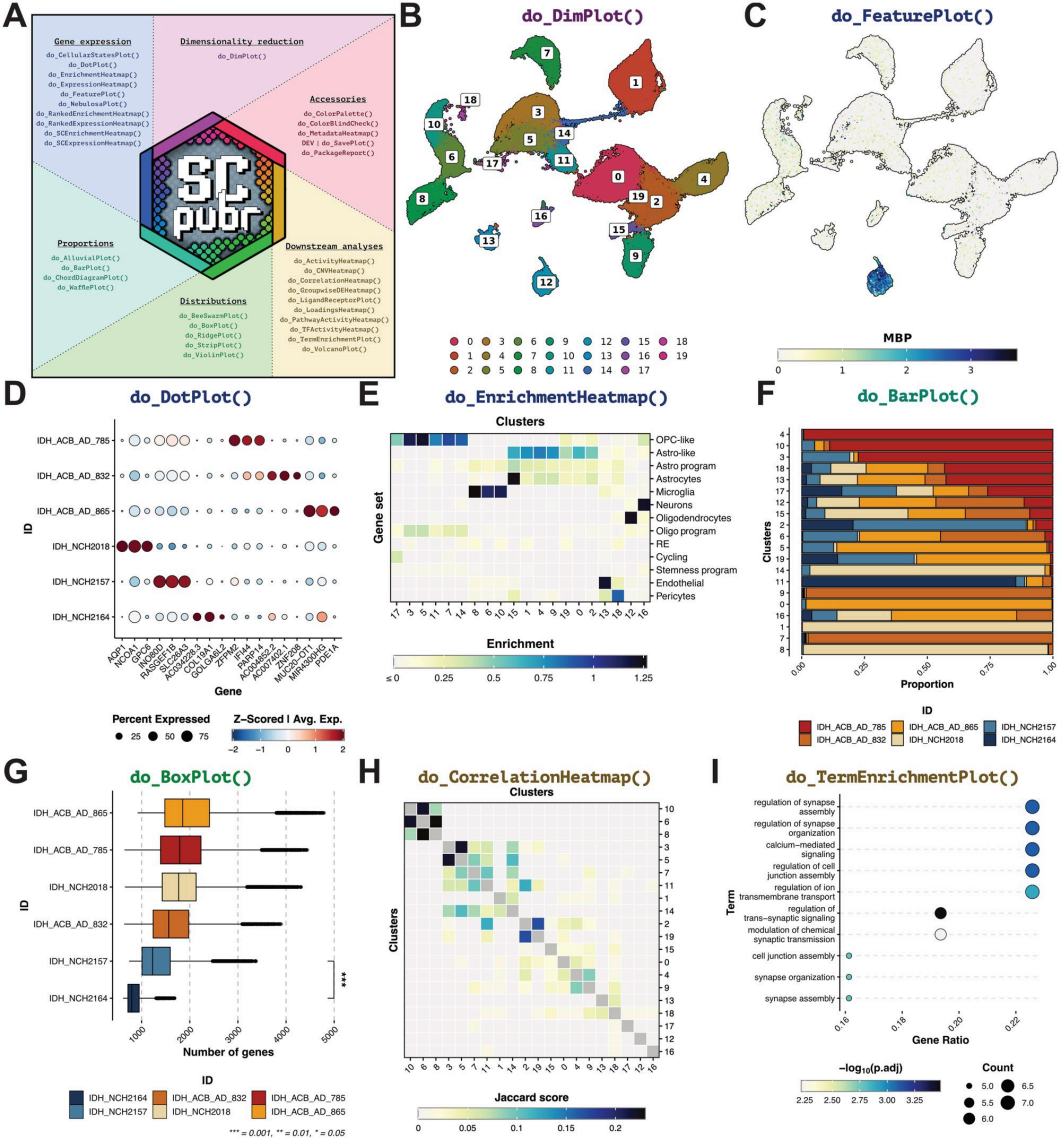
Core features of SCpubr. (A) Overview of SCpubr functions by topic: dimensionality reduction (pink), gene expression (blue), proportions (cyan), distributions (green), downstream analyses (yellow) and accessories (red). Functions marked with “DEV |” are exclusively available via GitHub installation; all other functions shown are available in the CRAN release. (B, C) Uniform Manifold Approximation and Projection (UMAP) embedding where cells are colored based on the results of clustering (B) and by Myelin Basic Protein (MBP) expression levels (C). UMAP1, *x*-axis. UMAP2, *y*-axis. (D) Dot plot showing the *z*-scored expression of the top three differentially expressed genes for each tumor patient. Color encodes for expression and size for percentage of cells within each patient expressing the gene. (E) Heatmap displaying the enrichment of cell clusters in various annotation gene sets reported by Blanco-Carmona *et al*. 2023. Clusters, *x*-axis. Gene sets, *y*-axis. (F) Bar plot showing the tumor patient composition across cell clusters. Clusters are ordered by descending proportions of tumor patient IDH_ACB_AD_785. Proportion, x-axis. Cell clusters, y-axis.(G) Box plot illustrating the distribution of the number of genes across tumor patients. Statistically significant differences between patients IDH_2157 and IDH_2164 were observed (Wilcoxon test, *P* value < 0.001). Number of genes, *x*-axis. Tumor patients, *y*-axis. (H) Heatmap correlating the Jaccard similarity between the top 250 differentially expressed genes for each cell cluster. Higher similarity score denotes a higher proportion of shared genes between a given pair of gene sets. (I) Dot plot illustrating the top 10 enriched GO terms for the Neurons annotation gene set reported by Blanco-Carmona *et al*. 2023. Color encodes for the statistical significance, while size does for the number of genes supporting each term. Gene ratio, *x*-axis. GO term, *y*-axis.

SCpubr provides a collection of functions designed to streamline the figure generation at multiple stages of single-cell transcriptome analysis. The primary input for most functions is a Seurat object, which serves as a standardized container for expression data, cell metadata, and dimensional reductions, with a subset of functions accepting tool-specific outputs (Table S2, available at supplementary material  *Bioinformatics Advances* online). These functions can be broadly categorized into six main topics: dimensionality reduction, gene expression, proportions, distributions, downstream analyses, and accessories (Fig 1A). To illustrate SCpubr’s capabilities, we used a subset comprising six IDH1-mutant astrocytomas (72,385 nuclei) from the publicly available dataset from Blanco-Carmona et al. (GSE20771) (Blanco-Carmona *et al.* 2023), replicating the original analysis methods and utilizing annotation resources as provided by the authors.

### 2.1 Dimensionality reduction

A central step in analyzing single-cell transcriptome data is dimensionality reduction, which reduces dataset complexity by identifying components that capture the main sources of variability. These components can then be used for visualization, enabling researchers to explore the underlying structure of the data (Heumos *et al*. 2023). Visualizing cells in this reduced space based on specific dimensional reduction components is essential for understanding the major biological factors driving the heterogeneity of the dataset (Fig. 1B). SCpubr visualizes precomputed reductions stored in the Seurat object, accessible via *Seurat::Reductions()*. For multi-dimensional reductions such as Principal Component Analysis (PCA), users can select which dimensions to visualize.

### 2.2 Gene expression

A cornerstone of single-cell transcriptomic analyses is the ability to examine gene expression across different cell populations and conditions. This is essential not only for identifying and annotating subsets of cells but also for performing meaningful comparisons. Common strategies include visualizing expression values (Fig. 1C) or expression densities (Fig. S1A, available at supplementary material  *Bioinformatics Advances* online) in the context of a dimensional reduction, as well as inspecting expression levels (Fig. 1D, Fig. S1B-C, available at supplementary material  *Bioinformatics Advances* online) or enrichment scores (Fig. 1E, Fig. S1D, available at supplementary material  *Bioinformatics Advances* online) across groups of cells. Certain dimensionality reduction methods, such as diffusion maps, are particularly effective for ordering cells along trajectories defined by specific gene sets, enabling visualization of gene expression (Fig. S1E, available at supplementary material  *Bioinformatics Advances* online) or enrichment values (Fig. S1F, available at supplementary material  *Bioinformatics Advances* online) along these axes. Comparisons across multiple gene sets are often critical for addressing biological questions. Visualization approaches can represent cells in the context of two (Fig. S1G-left, available at supplementary material  *Bioinformatics Advances* online), three (Fig. S1G-middle, available at supplementary material  *Bioinformatics Advances* online), or four (Fig. S1G-right, available at supplementary material  *Bioinformatics Advances* online) distinct gene sets. Established strategies for comparing enrichment in three and four distinct gene sets were introduced by Tirosh *et al*. (2016) and Neftel *et al*. (2019), respectively.

### 2.3 Proportions

Understanding how cells are grouped and categorized within a dataset is essential for accurate annotation and for enabling statistical comparisons of cell-type proportions across conditions. Proportions can be visualized using bar plots (Fig. 1F), where bars may be ordered by descending proportions of a given category within a group, or waffle plots (Fig. S2A, available at supplementary material  *Bioinformatics Advances* online), where proportions are represented as a grid of 100 tiles, each corresponding to one percent of the total. To illustrate shifts in proportions between categories, alluvial plots (Fig. S2B, available at supplementary material  *Bioinformatics Advances* online) or chord diagram plots (Fig. S2C, available at supplementary material  *Bioinformatics Advances* online) can be used, with the former displaying changes sequentially and the latter in a circular layout.

### 2.4 Distributions

Some research questions require analyzing the distribution of gene expression across categorical groups to derive biological insights. Several visualization approaches are available for this purpose. Box plots (Fig. 1G) provide a summary of a distribution spread and outliers, while violin plots (Fig. S2D, available at supplementary material  *Bioinformatics Advances* online) emphasize distribution shape and density. Ridge plots (Fig. S2E, available at supplementary material  *Bioinformatics Advances* online) are particularly effective for highlighting patterns across groups, especially when categories follow a sequential order. At the per-cell level, strip plots (Fig. S2F, available at supplementary material  *Bioinformatics Advances* online) represent data as a one-dimensional scatterplot, aligning points along an axis for each category. Similarly, beeswarm plots (Fig. S2G, available at supplementary material  *Bioinformatics Advances* online) arrange points to avoid overlap, providing a clearer overview of distribution density.

### 2.5 Downstream analyses

Single-cell transcriptomic datasets enable a broad range of downstream analyses, each addressing distinct biological questions. Visualizing these outputs is critical for drawing biological conclusions. For example, correlations between gene sets can be quantified using Jaccard similarity and displayed as a heatmap (Fig. 1H), while differentially enriched Gene Ontology (GO) terms are often represented as a dot plot (Fig. 1I). Heatmaps are also effective for inspecting activity scores generated with decoupleR (Badia-I-Mompel *et al*. 2022), whether based on custom prior knowledge networks (Fig. S3A, available at supplementary material  *Bioinformatics Advances* online), curated cancer pathway gene sets (Fig. S3B, available at supplementary material  *Bioinformatics Advances* online), or transcription factors and their downstream targets (Fig. S3C, available at supplementary material  *Bioinformatics Advances* online). Differential expression can be visualized across multiple groups with heatmaps (Fig. S3D, available at supplementary material  *Bioinformatics Advances* online) or between pairs of categories with volcano plots (Fig. S3E, available at supplementary material  *Bioinformatics Advances* online). Additional downstream analyses and visualizations include copy number variant (CNV) analyses with scores per chromosome arm (computed with inferCNV (inferCNV of the Trinity CTAT Project., n.d.)) as a heatmap (Fig. S3F, available at supplementary material  *Bioinformatics Advances* online), visualizing ligand-receptor interaction scores across groups from liana (Dimitrov *et al*. 2022) as a dot plot (Fig. S3G, available at supplementary material  *Bioinformatics Advances* online), and pairing principal component loadings with the expression of their top contributing genes in a heatmap (Fig. S3H, available at supplementary material  *Bioinformatics Advances* online). A detailed mapping of upstream tools, expected input formats, and corresponding SCpubr functions for all downstream visualizations is provided in Table S2, available at supplementary material  *Bioinformatics Advances* online.

### 2.6 Accessories

Beyond the data itself, several additional aspects are crucial for effective visualization. These include displaying dataset metadata as a categorical heatmap (Fig. S4A, available at supplementary material  *Bioinformatics Advances* online) and maintaining a consistent, visually appealing color palette across figures and categories (Fig. S4B, available at supplementary material  *Bioinformatics Advances* online). Ensuring that color palettes are colorblind-safe is particularly important for maximizing accessibility and scientific outreach (Fig. S4C, available at supplementary material  *Bioinformatics Advances* online).

To this end, SCpubr also provides alternative, literature-recommended (Wong 2011, Krzywinski, n.d., Color Universal Design (CUD)/Colorblind Barrier Free, n.d., Paul Tol’s Notes, n.d.) default colorblind-safe palettes across all functions with categorical color scales, automatically selected based on the number of categories through the colorblind = TRUE parameter. Finally, since all SCpubr functions return standard ggplot2 (ggplot2: Elegant Graphics for Data Analysis (3e), n.d.) objects, figures can be exported to any format using *ggplot2::ggsave()*. For functions with continuous and divergent color scales, colorblind-safe palettes (YlGnBu and RdBu, respectively) are used as defaults without requiring any additional user input. Additionally, SCpubr offers a seamless export solution to multiple formats simultaneously via the *do_SavePlot()* function, currently available only through the GitHub installation. This solution will remain GitHub-only, as CRAN policies do not allow for functions that write directly into the user’s file system. This is the only difference between CRAN and GitHub version.

## 3 Implementation and reproducibility

SCpubr is implemented in R (≥ 4.0.0) and available on CRAN. All figures in this manuscript were generated using the CRAN release (v3.0.0) and Seurat v5. SCpubr is compatible with both Seurat v4.x and v5.x. The primary input for most functions is a Seurat object, with a subset of functions accepting tool-specific outputs (Table S2, available at supplementary material  *Bioinformatics Advances* online). All functions return ggplot2 objects, which can be further customized using standard ggplot2 syntax. Individual panels in this manuscript were exported via do_SavePlot(), available only through the GitHub development version. When using the CRAN version, users can utilize *ggplot2::ggsave()* instead. Panels were then composed using Affinity Designer. Programmatic assembly via patchwork is also supported. Key dependencies of SCpubr, necessary to run all functions include: Seurat, dplyr (Wickham *et al*. 2026), ggplot2 and patchwork (Pedersen 2025). A comprehensive summary of SCpubr’s dependencies can be inspected with *SCpubr::do_PackageReport()*. Full session information and a minimal reproducible workflow are provided in the SCpubr-manuscript repository (https://github.com/enblacar/SCpubr-manuscript).

## 4 Limitations

Certain visualization types have practical readability constraints. Alluvial plots and chord diagrams become difficult to interpret beyond approximately 10–15 categories, and metadata heatmaps may appear crowded with many simultaneous annotations. Figure readability ultimately depends on the complexity of the data being displayed. Users are responsible for curating their inputs (i.e., subsetting categories or selecting representative annotations) to ensure clear, interpretable figures. SCpubr assists this process by providing automatic group reordering, hierarchical clustering, and, for scatter-based plots, rasterization, point shuffling, and density contour overlays. Performance may be compromised for very large datasets.

## Supplementary Material

vbag151_Supplementary_Data

## Data Availability

SCpubr is available on CRAN (https://cran.r-project.org/package=SCpubr), with source code accessible on GitHub (https://github.com/enblacar/SCpubr). Extensive documentation and tutorials are available via the GitHub Pages site (https://enblacar.github.io/SCpubr-book/). The complete analysis code used to generate all figures in this publication, along with the full R session information and instructions for obtaining the raw input data, is available in GitHub (https://github.com/enblacar/SCpubr-manuscript).
