# Supplementary material for: SCpubr: a user-friendly R-package for generating publication-ready visualizations of single-cell transcriptome analyses
Source: Bioinform Adv. 2026 Jun 20;6(1):vbag151. doi: 10.1093/bioadv/vbag151 (PMC13282709; doi:10.1093/bioadv/vbag151)
Supplement: vbag151_Supplementary_Data [file vbag151_supplementary_data.zip › Blanco_Carmona_Supplementary_Figure_and_Table_Legends.docx]

**Supplementary Table 1:** Systematic comparison of the scope, intended use, input structure, design philofophy, and availability of different visualization types between SCpubr (v3.0.0) and Seurat (v5.4.0), dittoSeq (v1.21.1), scCustomize (v3.2.4), LotOfCells (v0.1.4), plot1cell(0.0.0.9000), iSEE (v2.23.1). Figure panel is provided for reference, mapping each feature to the respective figure panel in the manuscript. “-“ denotes that the feature is not available.

**Supplementary Table 2:** SCpubr functions, upstream tools, and expected input formats for all figure panels in this manuscript.

**Supplementary Figure 1: Extended gene expression.** **(A)** UMAP embedding depicting MBP expression density across cells. UMAP1, x-axis. UMAP2, y-axis. **(B)** Heatmap showing averaged expression levels of the top three differentially expressed genes for each tumor patient. Genes, x-axis. Patient, y-axis. **(C-D)** Heatmap of a representative subset of 200 cells per tumor patient, showing the expression of the top ten Microglia markers (C) and the enrichment in annotation gene sets **(D)** reported by Blanco-Carmona *et al*.^1^. Subset of cells per patient, x-axis. Genes / gene sets, y-axis. **(E-F)** Heatmap of a representative subset of 500 cells across all tumor patients ordered by PCA principal component 1 (PC1), showing the z-scored expression of the top ten Microglia markers **(E)** and the z-scored enrichment in the annotation gene sets **(F)** reported by Blanco-Carmona *et al*.^1^. Cells, x-axis. Genes / gene sets, y-axis. **(G)** Scatter plots where cell position is based on enrichment in multiple gene sets. For two genes sets **(left)**, enrichment is mapped to x- and y-axes. For three **(middle)** and four **(right)** gene sets, positions are calculated using the methods of Tirosh *et al*.^2^ and Neftel *et al*.^3^, respectively.

**Supplementary Figure 2: Extended proportions and distributions.** **(A)** Waffle plot representing the patient composition of cells as a 10x10 grid, where each tile correspond to 1% of the total cells. Colors encode for tumor patients. **(B)** Alluvial plot showing shifts in cell proportions across cell clusters and tumor samples. The size of the boxes is proportional to the number of cells in each group. Groups, x-axis. Cells, y-axis. **(C)** Chord diagram illustrating the proportion of cells from each tumor patient contributing to each cell cluster. The width of the arrows represents the proportion of cells shared between groups. **(D-G)** Distribution of the total number of genes per cell and tumor patient shown through various plot types: violin plot **(D)** ridge plot **(E)**, strip plot **(F)** and beeswarm plot **(G)**. Number of genes, x-axis. Tumor patients, y-axis.

**Supplementary Figure 3: Extended downstream analyses.** **(A-C)** Heatmaps showing activity scores across cell clusters in three contexts: annotation gene sets reported by Blanco-Carmona *et al*.^1^ (add reference) **(A)**, common cancer pathways **(B)**, and regulons (transcription factors and their downstream targets) **(C)**. Clusters: x-axis. Gene set / pathway / regulon: y-axis. **(D)** Dot plot illustrating the average expression levels of the top four differentially expressed genes for each tumor patient. Additional tiles encode for significance (-log_10_(p.adj.)) and magnitude (avg_log_2_(FC)). Genes, x-axis. Patients: y-axis. **(E)** Volcano plot showing differentially expressed genes between two cell clusters. Top three differentially expressed genes for each condition are labelled. Avg.log2(FC), x-axis. -log10(p.adj.), y-axis. **(F)** Heatmap showing averaged CNV scores for each chromosome arm and cell cluster derived from inferCNV analysis. Chromosome arm, x-axis. Cluster: y-axis. **(G)** Dot plot visualizing the specificity and magnitude of ligand-receptor interactions between selected cell clusters. Dot color represents expression magnitude and dot size represents specificity. Clusters: x-axis. Interactions, y-axis. **(H)** Heatmap displaying the top five and bottom five PCA loading genes for the first three principal components (PCs). Color in the top heatmap encodes for the loading score, while in the bottom heatmap encodes for the average expression of each gene per cell cluster. Gene, x-axis. Cluster / PC: y-axis.

**Supplementary Figure 4: Accessories.** (**A**) Metadata heatmap visualizing tumor patient variables, including ATRX status, diagnosis, tumor grade and gender. (**B**) Diagram demonstrating hue color variations while maintaining identical brightness and contrast values. (**C**) Heatmap visualizing the three most common colorblindness types for a chosen color palette.

**References**

1. Blanco-Carmona, E., Narayanan, A., Hernandez, I., Nieto, J.C., Elosua-Bayes, M., Sun, X., Schmidt, C., Pamir, N., Özduman, K., Herold-Mende, C., et al. (2023). Tumor heterogeneity and tumor-microglia interactions in primary and recurrent IDH1-mutant gliomas. Cell Rep. Med., 101249. https://doi.org/10.1016/j.xcrm.2023.101249.

2. Tirosh, I., Venteicher, A.S., Hebert, C., Escalante, L.E., Patel, A.P., Yizhak, K., Fisher, J.M., Rodman, C., Mount, C., Filbin, M.G., et al. (2016). Single-cell RNA-seq supports a developmental hierarchy in human oligodendroglioma. Nature *539*, 309–313. https://doi.org/10.1038/nature20123.

3. Neftel, C., Laffy, J., Filbin, M.G., Hara, T., Shore, M.E., Rahme, G.J., Richman, A.R., Silverbush, D., Shaw, M.L., Hebert, C.M., et al. (2019). An Integrative Model of Cellular States, Plasticity, and Genetics for Glioblastoma. Cell *178*, 835-849.e21. https://doi.org/10.1016/j.cell.2019.06.024.
