# Supplementary figures and images for: SCpubr: a user-friendly R-package for generating publication-ready visualizations of single-cell transcriptome analyses
Source: Bioinform Adv. 2026 Jun 20;6(1):vbag151. doi: 10.1093/bioadv/vbag151 (PMC13282709; doi:10.1093/bioadv/vbag151)

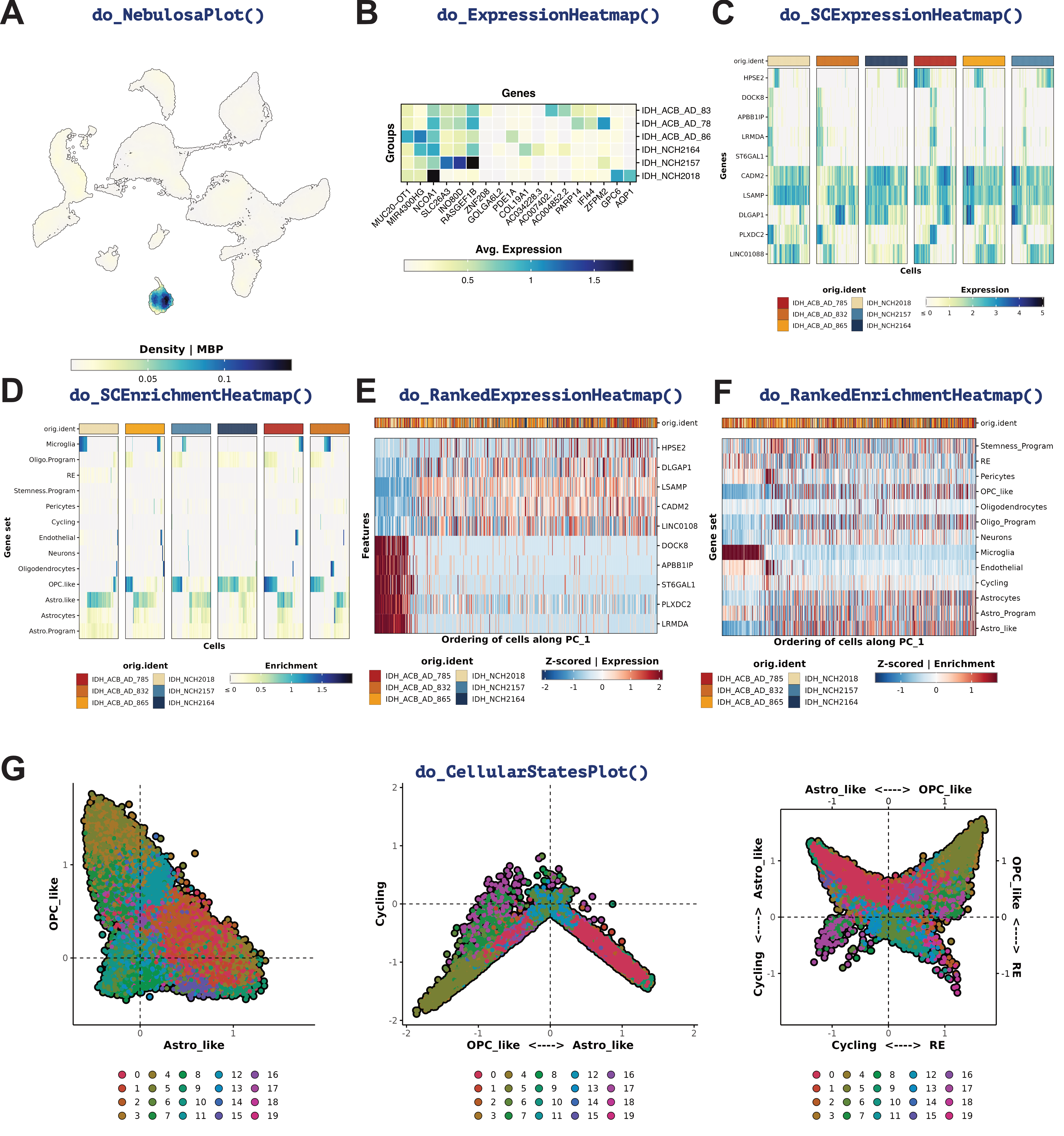

Supplement: vbag151_Supplementary_Data [file vbag151_supplementary_data.zip › Figure_S1.tiff]

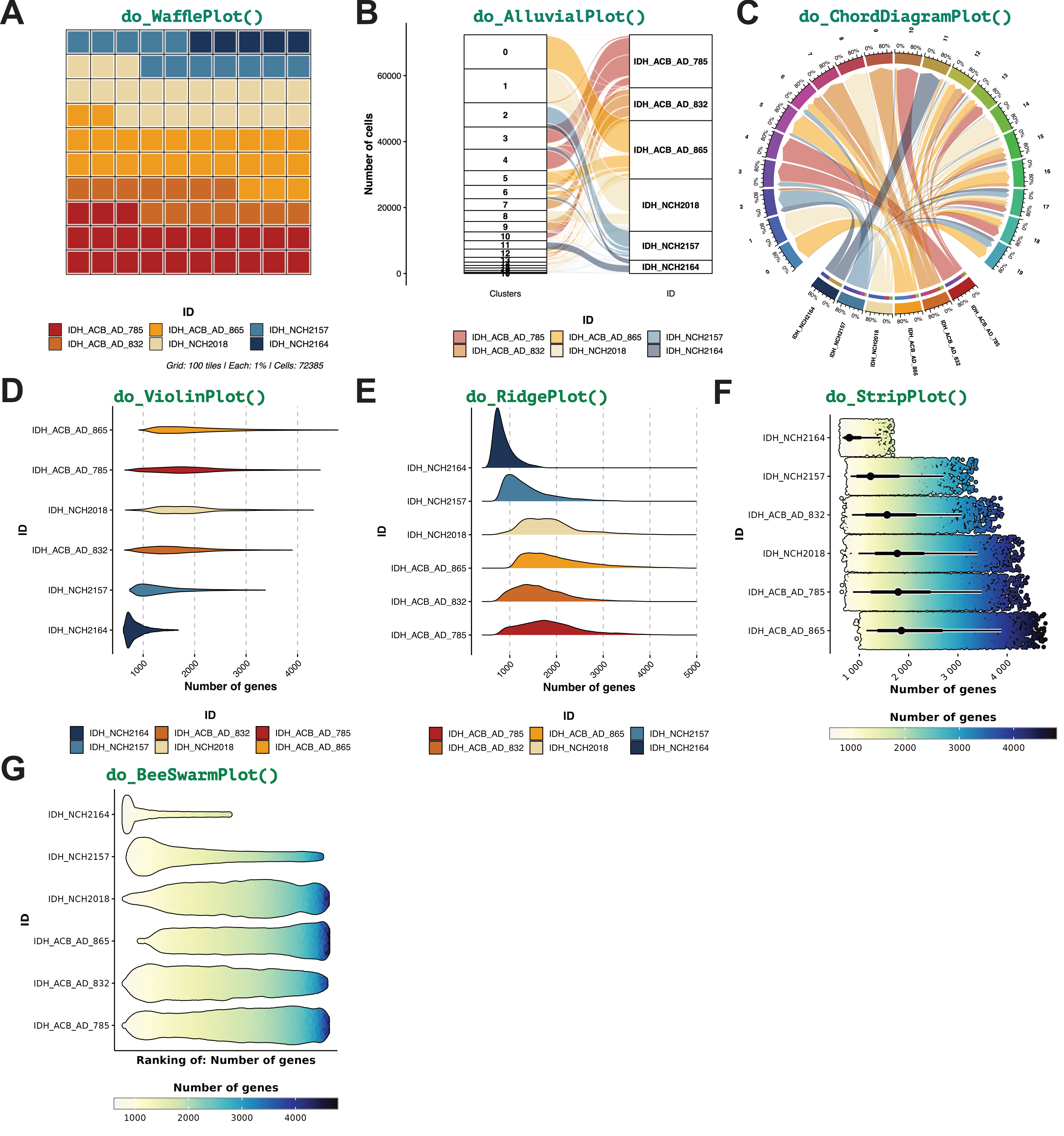

Supplement: vbag151_Supplementary_Data [file vbag151_supplementary_data.zip › Figure_S2.tiff]

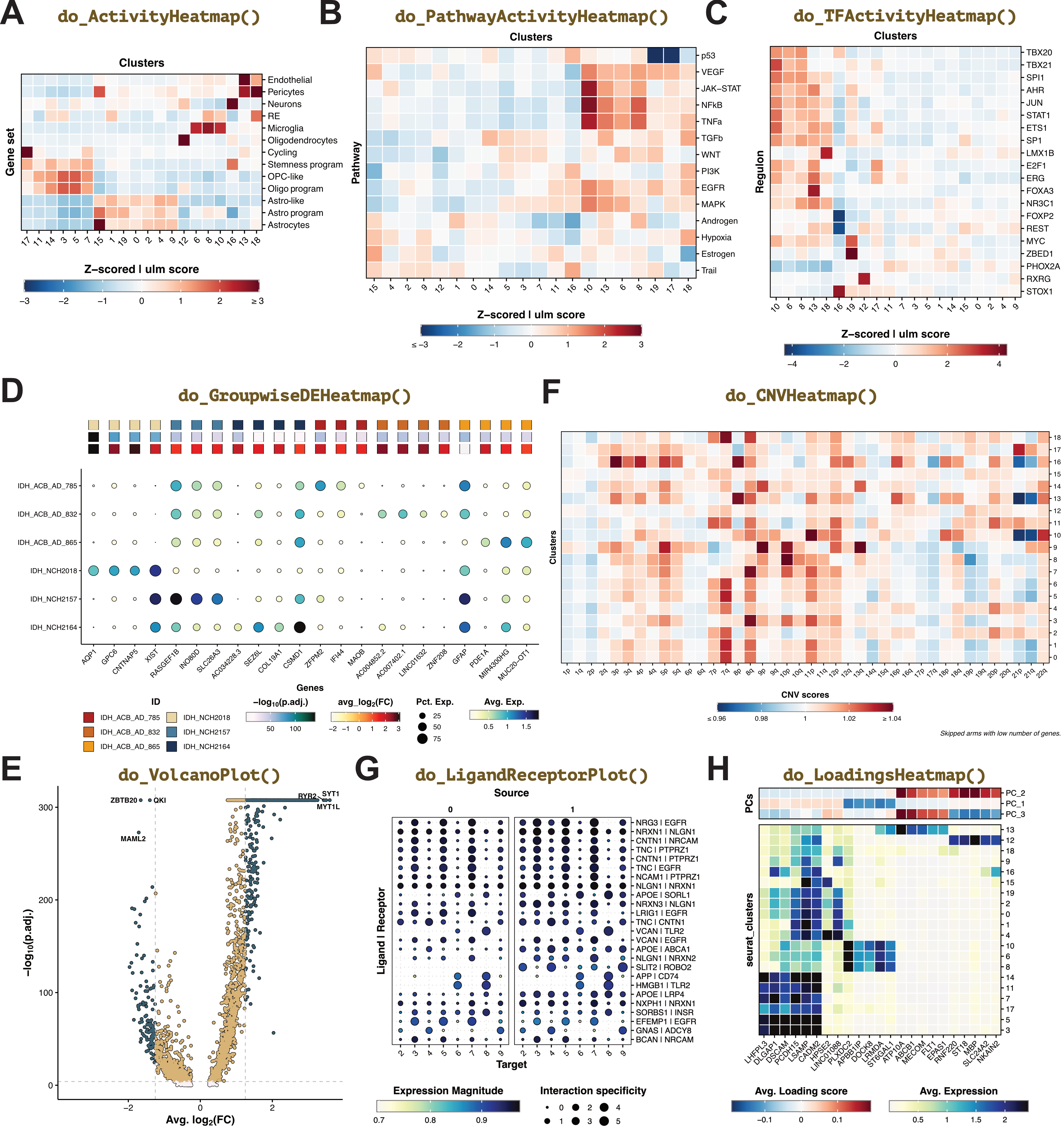

Supplement: vbag151_Supplementary_Data [file vbag151_supplementary_data.zip › Figure_S3.tiff]

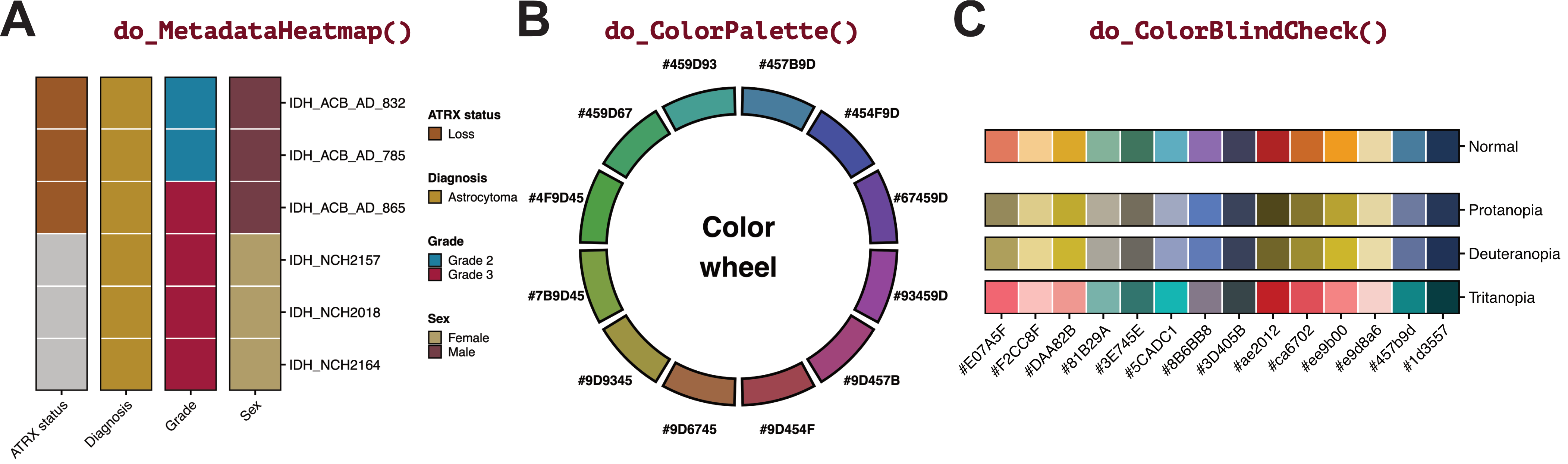

Supplement: vbag151_Supplementary_Data [file vbag151_supplementary_data.zip › Figure_S4.tiff]
